# Which is the most powerful adverse factor for autogenous access patency between diabetes and high arterial calcification burden?

**DOI:** 10.1080/0886022X.2018.1497518

**Published:** 2018-10-03

**Authors:** George S. Georgiadis, Christos Argyriou, Konstantia Kantartzi, Efstratios I. Georgakarakos

**Affiliations:** a Department of Vascular Surgery, “Democritus” University of Thrace, University General Hospital of Alexandroupolis, Alexandroupolis, Greece;; b Department of Nephrology, “Democritus” University of Thrace, University General Hospital of Alexandroupolis, Alexandroupolis, Greece

Dear Editor,

We have read with great interest the article by Yan et al. [1] demonstrating significantly increased risk of arterio-venous fistula (AVF) failure in diabetic by 1.7 times compared to nondiabetics patients. However, analyzing Asian and Caucasian population together may not be applicable due to inconsistent patient population. Additionally, the authors should mention in their study limitations including age, gender, type of dialysis population included (incident/prevalent), surgical site or technique, and operator experience. The fact that these additional factors were not investigated, whereas some of them are correlated with fistula blood vessel calcification is an important parameter that could be thoroughly explained in the meta-analysis.

Older patients have a higher incidence of peripheral vascular disease, atherosclerosis, hypertension, and arterial calcification. High prevalence of medial vascular calcification in fistula blood vessels was found in 75% of diabetic males with end-stage renal disease (ESRD) [2]. Diabetics had a 3.43-fold risk for vascular access calcification compared with nondiabetics [3]. Furthermore, regardless of the presence of ESRD, vascular calcification is found more frequently in male patients [4]. Others found that failure of the radiocephalic (RCF) fistula was more likely in women and diabetic patients [5]. Surprisingly, except race, no other demographics of the population studied were reported in this meta-analysis.

Calcification process progresses as dialysis vintage increases, and this is confirmed by the lower incidence of vascular access calcification in incidence patients (38% [6], 40% [7], 54% [8]) compared to higher values observed in prevalent patients (62% [2]). Ideally, this meta-analysis should distinguish incident/prevalent patients for better evaluation of the results.

Although, there is a complex interaction of factors that may affect the patency of an individual AVF, several prospective studies reported reduced effect of diabetes on primary access survival, in proximal AVFs [9,10] suggesting that AVF location predicts outcomes. Thus, should wrist, mid-antebrachial, and elbow fistulae be grouped together as the authors did? So how should the findings of this meta-analysis influence contemporary clinical practice if not stratified by AVF site? Which AVF location is worse for diabetics? We have shown that ESRD diabetics with pre-existing radial artery Mönckeberg macrocalcifications receiving RCF had worse late clinical outcomes (primary/secondary patency rates) compared with ESRD diabetics with healthy distal arm vessels receiving the same access. Others found also a positive association between arterial microcalcification and one year primary unassisted patency [6]. Therefore, in a diabetic population, primary RCF does not work equally between patients with highly calcified arteries and patients with healthy arteries [8].

Whether diabetes is a more powerful adverse factor of access patency compared to arterial calcification burden in diabetics receiving autogenous fistulas should be further investigated. Thus, should we need another meta-analysis distinguishing AVFs with medial calcification versus AVFs without medial calcification in diabetics? Notably, if the long-term benefit of RCF is lost, it may be prudent to select the brachial artery for AVF creation in diabetics with extensively calcified vessels [8]. Furthermore, Mōnckeberg’s sclerosis displayed in peripheral arteries of diabetics with ESRD, may reach such extend that when hemodynamically significant, hinders the maturation process by preventing the compensatory hypertrophy of the feeding artery and the subsequent increase in the arterial flow [11]. As a result, high flow rates are not achieved in these atherosclerotic distal feeding arteries, also subject to impaired vasodilatation secondary to endothelial cell dysfunction. The creation of a primary RCF results in an increase in shear stress, which causes arterial dilatation in an attempt to return shear stress levels back to normal [12,13]. However, the linkage between high shear stress and vascular dilatation in severe vascular disease and diabetes, possibly does not exist [14]. A severely calcified vessel as in Mōnckeberg disease cannot follow the flow-mediated vasodilatation required for normalization of shear stress. A study examining medial fibrosis and microcalcification scores in patients with mature and non-maturing fistulas (all autogenous options) reported a trend towards greater microcalcification in non-maturing fistulas, but recognized that with a larger sample size this trend might have been significant [15]. Although this possible scenario could explain fistula non-maturation, it remains unclear, and a field of further investigation, whether microcalcifications, frequently observed in arteries used to create AVF [15], are equally clinically important as macrocalcifications in specific locations like the wrist. Thus, precise calcification scores are required to find the limit that jeopardizes fistula survival.

**Figure 1.**
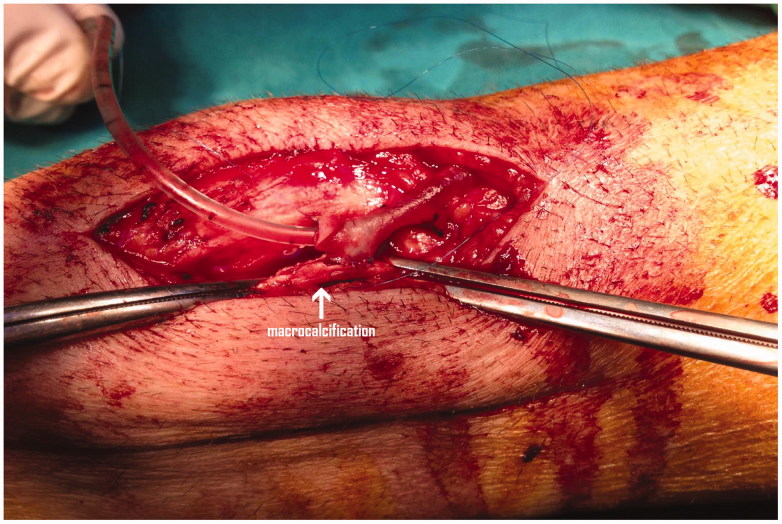
Creation of a radiocephalic fistula in a patient with severe radial artery wall calcifications (arrow).

It will be critical to carefully examine the vessel inflow and outflow of the AVF since the presence of small-diameter vessels at anastomosis sites is an important risk factor for AVF failure [16]. Previous studies have shown that vein size is a significant predictor of subsequent fistula survival [17], and that small vessel size predicted fistula failure in the first 3 months after surgery, respectively [16]. As such, the operating surgeon skill is implicated in the patency of autogenous fistulas and sufficient experience is required to overcome occasional technical problems like suturing a small vein to a severely calcified artery (Figure 1) and also to overcome other unexpected technical problems. Of interest, various 3-year patency rates have been reported (34–62%) depending on the vascular access surgeon, while a 2.4-fold increase of fistula failure is expected if the procedure is performed by unsupervised trainee surgeons [18]. Unfortunately the authors did not mention this important issue.

Based on the above described views and anatomic restrictions regarding fistula construction in diabetics with calcified forearm arteries, strong evidence is required before a profound change in strategy in this cohort of patients. In other words, it is not a simple meta-analysis question of AVF failure in diabetics versus nondiabetics in ESRD. As such, all the above factors constitute limitations of this study and at least deserve adequate mention. Furthermore, other risk factors for fistula survival (adjuvant treatments, follow-up protocols, comorbidity score, intradialytic hypotension, medications, biohumoral markers of endothelial damage, genetic milieu) should not be overlooked.
